# Intensifying cropping sequences in the US Central Great Plains: an *in silico* analysis of a sorghum–wheat sequence

**DOI:** 10.3389/fpls.2025.1525128

**Published:** 2025-05-30

**Authors:** Lucia Marziotte, Ana J. P. Carcedo, Daniel Rodriguez, Laura Mayor, P. V. Vara Prasad, Ignacio A. Ciampitti

**Affiliations:** ^1^ Department of Agronomy, Kansas State University, Manhattan, KS, United States; ^2^ Center for Crop Sciences, Queensland Alliance for Agriculture and Food Innovation (QAAFI), The University of Queensland, Gatton, QLD, Australia; ^3^ Corteva Agriscience, Johnston, IA, United States; ^4^ Department of Agronomy, Purdue University, West Lafayette, IN, United States

**Keywords:** sorghum, wheat, cropping sequence, great plains, APSIM, modeling

## Abstract

**Introduction:**

In the Central Plains of the United States (US), wheat (*Triticum aestivum* L.) is predominantly grown as a monocrop, limiting profits, and compromising environmental sustainability. In the context of recent reports on crop yield stagnation and the increased frequency and intensity of climate extremes, this study aims to i) evaluate the economic feasibility of double cropping sorghum (*Sorghum bicolor* L.) with winter wheat; ii) identify regional environmental drivers for yield; and iii) map the spatial distribution of the most profitable crop sequences.

**Methods:**

The APSIM classic model was used to simulate the baseline wheat and sorghum monocrops and the diversified crop sequence (sorghum-wheat) over 30 years of climatology (1990 to 2020), across 194 sites in Kansas, United States. Each site was characterized in APSIM, with the predominant soil type and current farming crop management practices. Using terciles of historical input costs for all crop sequences we calculated three cost scenarios low, intermediate, and high. A fuzzy-C means algorithm was used to classify regions based on crop sequences’ profits, resulting in four clusters.

**Results and discussion:**

Results included two regions where sorghum-wheat was more profitable than the monocrops i.e., one with lower profits (S+W lower), and a second one with higher profits (S+W higher); a third cluster where wheat monocrop was most profitable (W), and lastly one cluster showing no difference between the sorghum-wheat sequence and the wheat monocrop (S+W or W). Principal component analyses were used to identify environmental drivers of profit in each cluster. Results showed that the profitability of the sorghum-wheat sequence was higher in counties in the south-east and south-central of Kansas. Wheat monocrops were the most profitable option for counties of the west and central regions. Counties from the north-east of the state showed similar patterns amongst scenarios. These results highlight potential avenues for diversifying and intensifying the current wheat monocrop sequence while maintaining or increasing profitability. Lastly, this study delineates a map in Kansas with areas where it would be more profitable for farmers to expand their rotations by adding a second crop per year.

## 1 Introduction

The Central Great Plains of the USA has the largest sown area of winter wheat (*Triticum aestivum* L.) monocrop in the globe (Fischer et al., 2014). Winter wheat is a well-adapted crop to the region’s growing conditions (high evaporative demand and limited annual rainfall) and is commonly grown as a monoculture (Deines et al., 2019). Monocropping is known to result in yield stagnation (Patrignani et al., 2014), soil degradation (Mikha et al., 2014), poor weed control, and diseases (Gebru, 2015; Liebman and Dyck, 1993). Kansas is the number one wheat-producing state in the USA (USDA-NASS, 2023), and wheat is grown mostly as a monocrop continuously from October to June, followed by a 4-month fallow period (Jaenisch et al., 2021; Massigoge et al., 2024). Including other crops in the crop sequence can increase economic returns, reduce abiotic and biotic risks, and deliver environmental sustainability outcomes (Carolan, 2016; Hoppe, 2017).

From an economic perspective, there is significant pressure to enhance and diversify economic outcomes, as farmers are increasingly dependent on off-farm income, subsidies, and incentives from government programs (Carolan, 2016; Hoppe, 2017). In addition, the increased reliance on external inputs has rendered farmers susceptible to price and market fluctuations, as evidenced by the recent spike in urea prices (USDA Foreign Agricultural Service, 2022). Diversified rotations can help mitigate risks and increase returns and environmental outputs (Vitale et al., 2020). The inclusion of sorghum (*Sorghum bicolor* L.) in the crop sequence may be a feasible option given its tolerance to water and heat stresses (Hadebe et al., 2017) and larger biomass production than wheat (Schlegel et al., 2018). However, over the last two decades, there has been a significant reduction in the planted area of sorghum, resulting in the halving of the US Sorghum Belt, relegating the crop to the more marginal areas (Mayor et al., 2023). The potential for including sorghum in a rotation with wheat remains largely unexplored, offering a promising solution to increase profits and manage risks. However, several factors require more information before the practice can be promoted, in particular, the duration of the sorghum crop growing season, i.e., the time to harvest and its dry-down (Ciampitti et al., 2022) and potential delays in the planting of the subsequent winter crop (Assefa et al., 2014). To address this, early planting of sorghum has been proposed as a solution, coupled with the development of new hybrids having improved chilling tolerance to achieve uniform emergence (Ostmeyer et al., 2020). However, the same authors found that early planting often resulted in an extended vegetative stage compared to regular planting, which did not optimize land use and timely field turnover for subsequent crops. Another approach involves the use of early-maturing hybrids (Marziotte et al., 2025) to better match crop-sensitive stages with more favorable environmental conditions (i.e., warmer weather). Nevertheless, early-maturing hybrids have a shorter growth cycle and lower yield potential (Kamoshita et al., 1998). The introduction of early-maturing sorghum can also allow for double cropping (e.g., sorghum–wheat), favoring crop diversification, improving ground cover and soil condition, and increasing cropping intensity and profits.

The aims of this study were to i) evaluate the economic feasibility of double cropping sorghum with wheat under contrasting cost scenarios, ii) understand the environmental drivers behind profitable cropping activities (i.e., monocrops and cropping sequences) in Kansas, and iii) map the spatial distribution of the most profitable crop sequences in Kansas, as the main wheat- and sorghum-producing region of the USA.

## 2 Materials and methods

**Figure 1:**
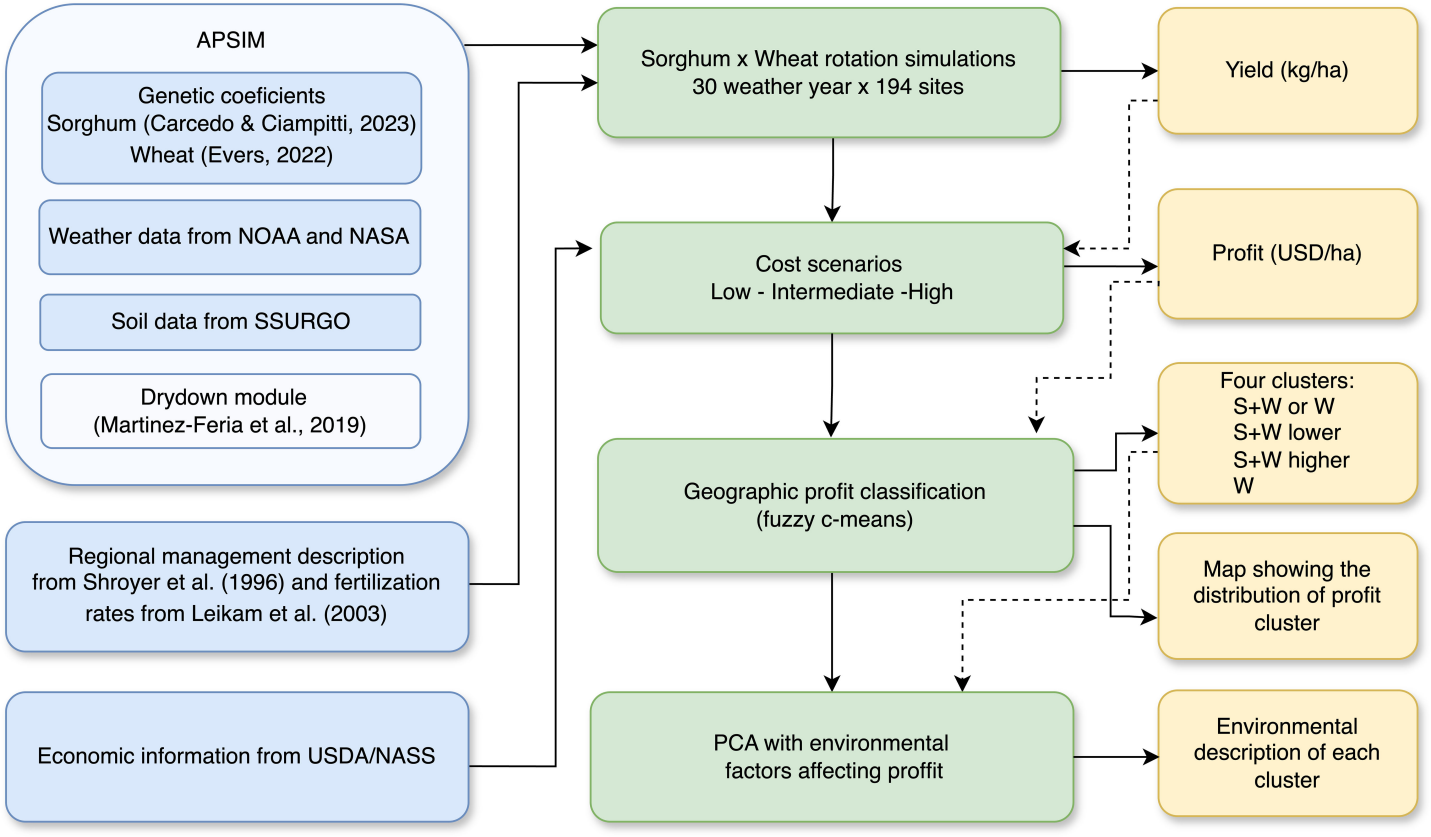
Diagram of the workflow from input sources through analytical procedures to final outputs. Blue boxes represent the data sources, green the process, and yellow the outputs. The data sources include genetic coefficients for sorghum and wheat for the APSIM model simulations, weather and soil data for each site and the dry-down module, Kansas regional management practices, and USDA/NASS economic data. As a first step, the simulation of sorghum monocrop, wheat monocrop, and sorghum–wheat crop sequence across 30 years and 194 sites was conducted. The output yields from the simulations were used to calculate profits across three different cost scenarios. These profits were then grouped using the fuzzy c-means clustering technique into four clusters to visualize the geographic distribution of the profits. Subsequently, a principal component analysis (PCA) was conducted for each cluster to evaluate the environmental factors influencing profitability.

A graphical description of the approach is presented in **Figure 1**. Briefly, the APSIM model (version 7.10) was used to simulate wheat and sorghum monocrops and a sorghum–wheat crop sequence across 194 sites in Kansas over 30 years of climatology. Profits were calculated for three cost scenarios (low, intermediate, and high). A fuzzy-C means algorithm was used on the simulated profits to classify regions based on the most profitable crop sequences.

### 2.1 APSIM simulations

APSIM is a widely used cropping system simulation model (Holzworth et al., 2014). More specifically, the APSIM sorghum module simulates phenology, crop growth, and development using a water demand and supply concept (Hammer et al., 2010). Similarly, APSIM wheat simulates winter and spring wheat daily growth and its phenological development (Zhao et al., 2014), responding to weather, soil water, soil nitrogen, and management (Zheng et al., 2015).

The APSIM model (version 7.10) was used to simulate three crop sequences: i) a monocrop sorghum, ii) a monocrop wheat, and iii) a sorghum–wheat crop sequence, i.e., two crops in the same year. Temperature and rainfall data spanning 30 years were obtained from NOAA for 194 sites in Kansas (National Oceanic and Atmospheric Administration, 2023). Radiation and relative humidity were obtained from NASA (National Aeronautics and Space Administration; NASA POWER, 2023) considering the latitude and longitude of the NOAA weather stations. All weather data were checked for errors such as the minimum temperature being lower than the maximum temperature for the day. Then, met files were created for each location and used in the simulations. The soil characteristics for each location, using the coordinates of the NOAA weather stations, were downloaded from SSURGO (Soil Survey Geographic Database; Soil Survey Staff, Natural Resources Conservation Service [NRCS], and United States Department of Agriculture [USDA], 2021), and the soil parameters were calculated employing the apsimx package (Miguez, 2022).

The simulations started 4 months before the sowing date to estimate the initial soil conditions. A sowing rule was used for both crops that included soil moisture availability and the sowing window. This is 30 mm of rainfall over three consecutive days needed to trigger a sowing event within a set sowing window. If the rule was not met, the sowing would occur on the last day of the sowing window. The sowing windows were defined based on the county recommendations (**Supplementary Table 1**) (Shroyer et al., 1996) for sorghum; the sowing window ranged from May 15 to June 25, depending on the location, and for wheat, the sowing window ranged from September 10 to October 25. In the case of the sorghum–wheat crop sequence, if sorghum had not reached harvest moisture before the last day of the wheat sowing window, the crop was harvested on that day, and the wheat was planted. Initial conditions, i.e., soil water and soil nitrogen, were reset at the start of each crop sequence. For the sorghum–wheat sequence, the simulations were ended after the wheat crop and reset for the next year. For the monocrops, the simulations ended after the harvest of each crop.

A dry-down module was incorporated into APSIM to simulate sorghum grain dry-down after maturity using Equation 1 (Martinez-Feria et al., 2019). Sorghum dry-down parameters were obtained from Paulsen and Thompson (1973). The validation of the dry-down module is shown in **Supplementary Figure 2**. The dry-down module assumed a 35% grain humidity at physiological maturity (Ciampitti et al., 2022) and triggered sorghum harvest at 14% humidity.


(1) $$\frac{dM}{dx}=\;-k\cdot \left(M-M_{e}\right)\cdot n\cdot x^{n-1}$$

where M is % grain humidity at the beginning of the day, Me stands for equilibrium moisture content, k stands for proportionality drying coefficient, and x is the days since maturity.

Two previously validated sorghum hybrids for KS (Carcedo and Ciampitti, 2023) were used. An early-maturing hybrid (Pioneer_s34) was used in the sorghum–wheat crop sequence, and a late-maturing hybrid (Buster) was used in the sorghum monocrop. The difference in maturity between the early- and late-maturing hybrids was 15 days (**Supplementary Figure 3**). The wheat variety ‘Larry’, previously validated in Kansas (Evers, 2022), was used in the wheat simulations. Crop coefficients are shown in **Supplementary Tables 2**–**4** for the two sorghum and wheat genotypes. Nitrogen fertilization requirements were calculated following the recommendations described in Leikam et al. (2003). This is considering the expected yield (averaged of 10 years; USDA-NASS, 2023) and assuming a soil organic matter content of 2.5%. Recommended plant density and sowing dates followed Shroyer et al. (1996). For wheat, sowing dates ranged from September 10 to October 25 and, for sorghum, from May 15 to July 10. Plant densities were based on site rainfall, with ranges from 1,488,120 to 2,777,820 plants per hectare for wheat, and 60,000 to 170,000 plants per hectare for sorghum.

### 2.2 Cluster analysis of profits

Profit calculations considered the cost of seed and fertilizer, which typically constitute 40% or more of the total input cost for both crops (Tsoodle and Li, 2023). Mean grain prices were obtained from the USDA/NASS (U.S. Department of Agriculture, National Agricultural Statistics Service) QuickStats *Ad-Hoc* Query Tool (quickstats.nass.usda.gov). For wheat, the reported prices for seed and fertilizer were considered representative of the central Kansas area; for the west and east areas, they were adjusted following seed and fertilization differences with central Kansas. Sorghum costs were determined based on the average for areas receiving between 508–660 and 660–812 mm of rain and adjusted to the rest of Kansas according to seed and fertilization rates. To provide a comprehensive economic analysis, three cost scenarios representing low-, intermediate-, and high-cost conditions were established. These scenarios were formulated using terciles of seed and fertilization costs spanning from 2014 to 2022 (available in **Supplementary Tables 5**, **6**). An average yield value was obtained from simulations and used to calculate gross income for the three cost scenarios. The profit for each cost scenario was obtained using a different grain price and different cost values.

### 2.3 Data analysis

The data analysis was conducted using the R software (R Core Team, 2021). A mean multiple comparison of the profit was performed between the cropping sequences and cost scenarios by fitting a linear model (package stats; R Core Team, 2021) and calculating the estimated marginal means (package emmeans; Rusell V. Rusell, 2024). Finally, the comparison between the profits was conducted using Sidak’s correlations (package multcomp; Hothorn et al., 2008). As a measure of risk, the variability in gross margins was calculated using the interquartile ratio (IQR) and standard deviation (SD) (stats package; R Core Team, 2021).

Regions having similar profits were spatially clustered. Four clusters were defined using the fuzzy C-means algorithm to classify sites based on the profits of the three cropping sequences and cost scenarios over 30 years. The number of clusters was decided based on the lowest within-cluster sum of squares, with a preference for a smaller number of defined regions (package factoextra; Kassambara and Mundt, 2020) (**Supplementary Figure 2**). The resulting clusters were named based on the cropping sequence showing the highest mean profit, i.e., S+W or W, S+W lower, S+W higher, and W. The profit for each cluster, cropping sequence, and cost scenario was analyzed using a mean multiple comparison of the profit between the clusters, cropping sequences, and cost scenarios by fitting a linear model (stats package; R Core Team, 2021) and calculating the estimated marginal means (package emmeans; Rusell V. Rusell, 2024). Finally, the comparison between the profits was conducted using Sidak’s correlations (package multcomp; Hothorn et al., 2008). As a measure of risk, the variability in gross margins was calculated using the interquartile ratio and standard deviation (stats package; R Core Team, 2021). Each county was assigned to a cluster based on their frequency of occurrence. If the difference between the most frequent cluster and the second most frequent cluster was less than 10%, the county was defined as the W+S or W cluster, which stands for no dominance of the sorghum–wheat crop sequence or wheat monocrop. The percentage of times that each cropping sequence presented higher profit within each cluster was also calculated.

A principal component analysis (PCA) was used to identify the association between environmental covariates and their relationship with profit. Environmental covariates included the simulated average of nitrogen deficit (nfact) and crop water deficit (swdef), i.e., the ratio between crop water demand and total plant available water during the growing season, and the cumulative incoming solar radiation (rad), average minimum temperature (min), average maximum temperature (max), cumulative thermal time (TT), and cumulative rain during the growing season (rain), as well as for the pre-flowering (from sowing to flowering) (rad_pre, min_pre, max_pre, TT_pre, and rain_pre) and post-flowering (from flowering to maturity) (rad_post, min_post, max_post, TT_post, and rain_post) periods. Finally, the number of days with temperatures higher than 37.4°C and 36°C (temp_high) and lower than 10°C and 0°C (temp_low) for sorghum and wheat, respectively, were also calculated (Brown et al., 2018; Prasad et al., 2015). The number of environmental covariates was reduced by eliminating highly correlated variables (higher than 0.8) (package stats; R Core Team, 2021). Only the principal components having eigenvalues higher than one were selected, and within these, the 10 variables showing the highest loading weights were selected (package factoextra; Kassambara and Mundt, 2020).

## 3 Results

### 3.1 Simulation yield analysis

Simulated sorghum yields were similar for the sorghum in the sorghum–wheat crop sequence and the monocrop scenarios (**Table 1**, **Supplementary Table 6**). However, simulations showed a reduction in wheat yield when wheat was grown after the sorghum crop, i.e., up to 60%. The sorghum–wheat crop sequence had higher profits than the other sequences in the low- and intermediate-cost scenarios but did not differ from wheat monocrop under the high-cost scenario (**Table 1**). Notably, the high-cost scenario showed the largest differences among the crop sequences, with profits of 581 USD ha^−1^ for sorghum, 733 USD ha^−1^ for wheat, and 731 USD ha^−1^ for sorghum–wheat. It is also noteworthy that the costs associated with the scenarios were correlated with the profits; i.e., grain prices and profit were higher for the high-cost scenario and lower for the low-cost scenario. Additionally, wheat monocrops showed the lowest variability in profit (IQR and SD) among the cost scenarios.

**Table 1:** Yield for each crop in the different cropping sequence, and profit, interquartile ratio (IQR), and standard deviation (SD) for each rotation and cost scenario.

Rotation	Wheat yield (kg ha^−1^)	Sorghum yield (kg ha^−1^)	Cost scenarios	Profit (USD ha^−1^)	IQR	SD
Sorghum–wheat rotation	1,369 a	3,857 a	Low	537 c	257	186
			Intermediate	567 d	271	196
			High	731 f	336	248
Sorghum monocrop		4,129 b	Low	460 a	253	177
			Intermediate	485 b	267	182
			High	581 e	320	223
Wheat monocrop	3,413 b		Low	449 a	174	145
			Intermediate	482 b	187	155
			High	733 f	279	232

Different letters mean a significant difference in the same column between the values of (p < 0.05) according to Sidak’s test.

### 3.2 Cluster analysis of profits

The profits obtained by different county-by-year combinations and cost scenarios were classified into four clusters based on the cropping sequence that had the highest profit. The sorghum–wheat crop sequence had higher profits in two clusters: one with higher incomes (S+W higher) and the other with lower incomes (S+W lower). A third cluster included instances where there was no difference between having the sorghum–wheat crop sequence or wheat monocrop (S+W or W), and the last cluster included instances where wheat monocrop had higher profits (W). Notably, the low- and intermediate-cost scenarios presented similar profits across clusters and cropping sequences (means of 454 and 481 USD ha^−1^, respectively; **Table 2**). In cluster S+W higher, it was consistently advantageous to opt for the sorghum–wheat rotation over sorghum or wheat in monocrop, irrespective of the cost scenario (mean across cost scenarios of 872, 729, and 637 USD ha^−1^, respectively; **Table 2**). In cluster S+W lower, the sorghum–wheat crop sequence was more profitable across cost scenarios, but the profits were lower (mean across cost scenarios of 662 USD ha^−1^ for sorghum–wheat, 590 USD ha^−1^ for sorghum, and 480 USD ha^−1^ for wheat; **Table 2**). In the case of the W cluster, planting wheat was consistently more profitable across all cost scenarios (a difference of 293 USD ha^−1^ with sorghum and 67 USD ha^−1^ with the sorghum–wheat crop sequence; **Table 2**).

**Table 2:** Profit, interquartile ratio, and standard deviation for each rotation, cluster, and cost scenario.

Rotation	Cluster	Cost scenario	Profit (USD ha^−1^)	IQR	SD
Sorghum–wheat rotation	S+W or W	High	393 gh	237	179
		Intermediate	300 e	193	143
		Low	283 de	181	134
	S+W lower	High	782 uv	202	152
		Intermediate	618 p	159	120
		Low	587 o	151	113
	S+W higher	High	1,036 y	202	151
		Intermediate	811 w	159	119
		Low	768 u	149	112
	W	High	653 qr	234	178
		Intermediate	492 k	185	138
		Low	463 j	174	130
Sorghum monocrop	S+W or W	High	282 de	232	170
		Intermediate	234 ab	190	141
		Low	222 a	177	132
	S+W lower	High	674 s	279	197
		Intermediate	563 n	230	162
		Low	535 lm	218	152
	S+W higher	High	832 x	312	216
		Intermediate	695 t	257	179
		Low	660 rs	242	168
	W	High	355 f	185	152
		Intermediate	295 de	153	125
		Low	280 de	145	118
Wheat monocrop	S+W or W	High	417 hi	347	207
		Intermediate	270 cd	232	139
		Low	252 bc	216	129
	S+W lower	High	636 pq	178	132
		Intermediate	417 i	120	89
		Low	388 g	112	83
	S+W higher	High	841 x	196	144
		Intermediate	554 mn	132	97
		Low	516 l	123	91
	W	High	797 wv	219	161
		Intermediate	524 l	147	108
		Low	489 k	137	101

Different letters mean a significant difference in the same column between the values of (p < 0.05) according to Sidak’s test. SD stands for standard deviation, and IQR stands for interquartile ratio. S+W or W means that there is no clear difference between wheat or sorghum–wheat crop sequence, S+W lower means the sorghum–wheat crop sequence presented higher profits and the income was lower, S+W higher means the sorghum–wheat crop sequence presented higher profits and the income was higher, and W means wheat monocrop presented higher profits.

For the S+W or W cluster, the sorghum–wheat crop sequence had a difference in profit of 13 USD ha^−1^ with wheat monocrop and 79 USD ha^−1^ with sorghum monocrop (**Table 2**). However, the main difference between crop sequences was observed when comparing the cost scenarios between the wheat–sorghum sequence and the wheat monocrop. In the low- and intermediate-cost scenarios, the sorghum–wheat sequence had higher profits than wheat monocrop (261 vs. 291 USD ha^−1^), and in the high-cost scenario, wheat monocrop had higher profits (417 vs. 393 USD ha^−1^). The standard deviation on profits and its interquartile ratio remained consistent across cropping sequences and clusters (from 83 to 216 USD ha^−1^ and from 112 to 347 USD ha^−1^, respectively; **Table 2**) and were consistently higher in the high-cost scenario than in low and intermediate (IQR was 235 vs. 174 USD ha^−1^ and SD was 170 vs. 126 USD ha^−1^ for high vs. intermediate and low, respectively).

### 3.3 Spatial distribution of clusters

**Figure 2:**
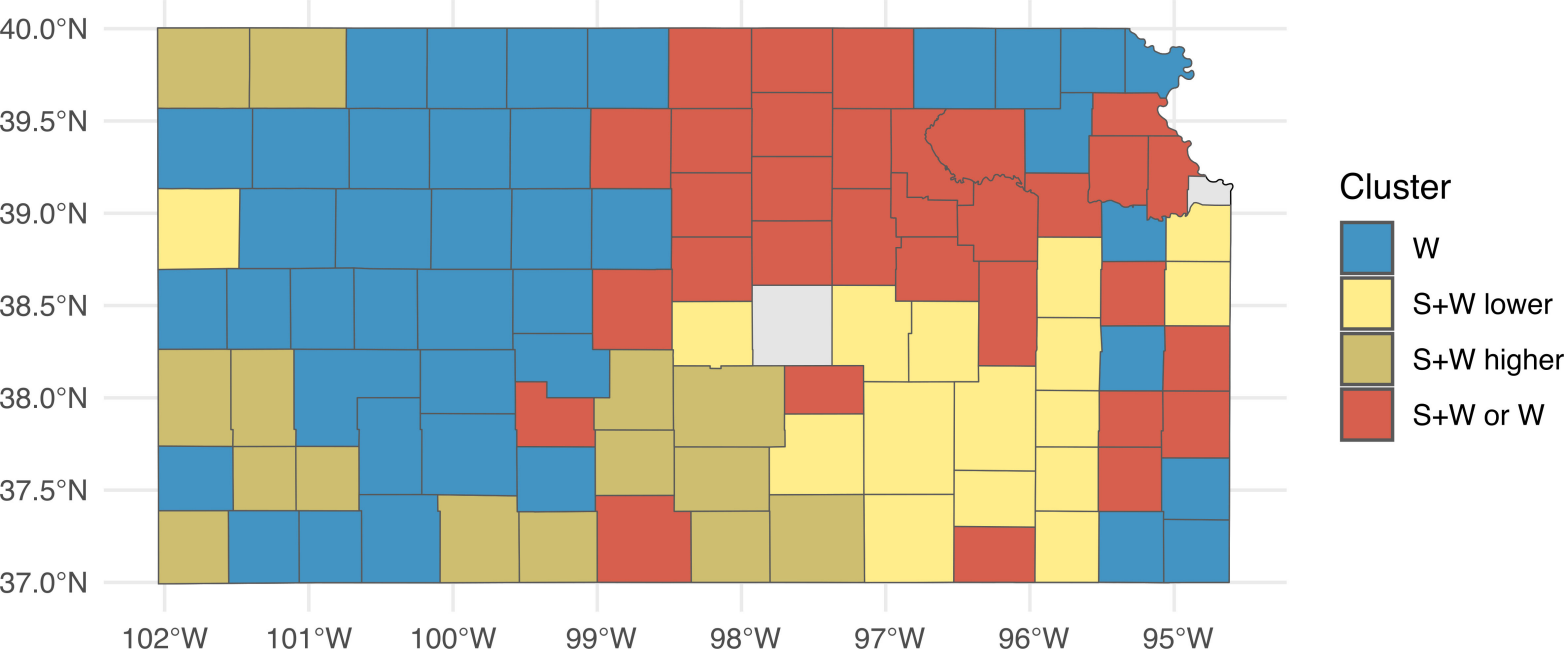
Map of the spatial distribution of the clusters. The different colors represent the different clusters. W means the county has a larger proportion of W cluster, which is the cluster in which wheat monocrop is the most profitable rotation; S+W lower means that the county has a larger proportion of S+W lower cluster, which is the cluster in which sorghum–wheat is the most profitable rotation and the profits are lower; S+W higher means the county has a larger proportion of S+W higher, which is the cluster in which sorghum–wheat is the most profitable rotation and the profits are higher; and S+W or W means that the county has a higher proportion of S+W or W, which is the cluster in which sorghum–wheat or wheat is the most profitable rotation or that no cluster is predominant in the county.

Cluster S+W or W was mostly located in the north-east area, S+W lower was predominantly found in the south-east of Kansas, and S+W higher was found next to S+W lower in the central-west areas of Kansas (**Figure 2**). Lastly, W concentrated in the western counties of the state. The cluster distributions had no spatial differences when comparing the three cost scenarios.

**Figure 3:**
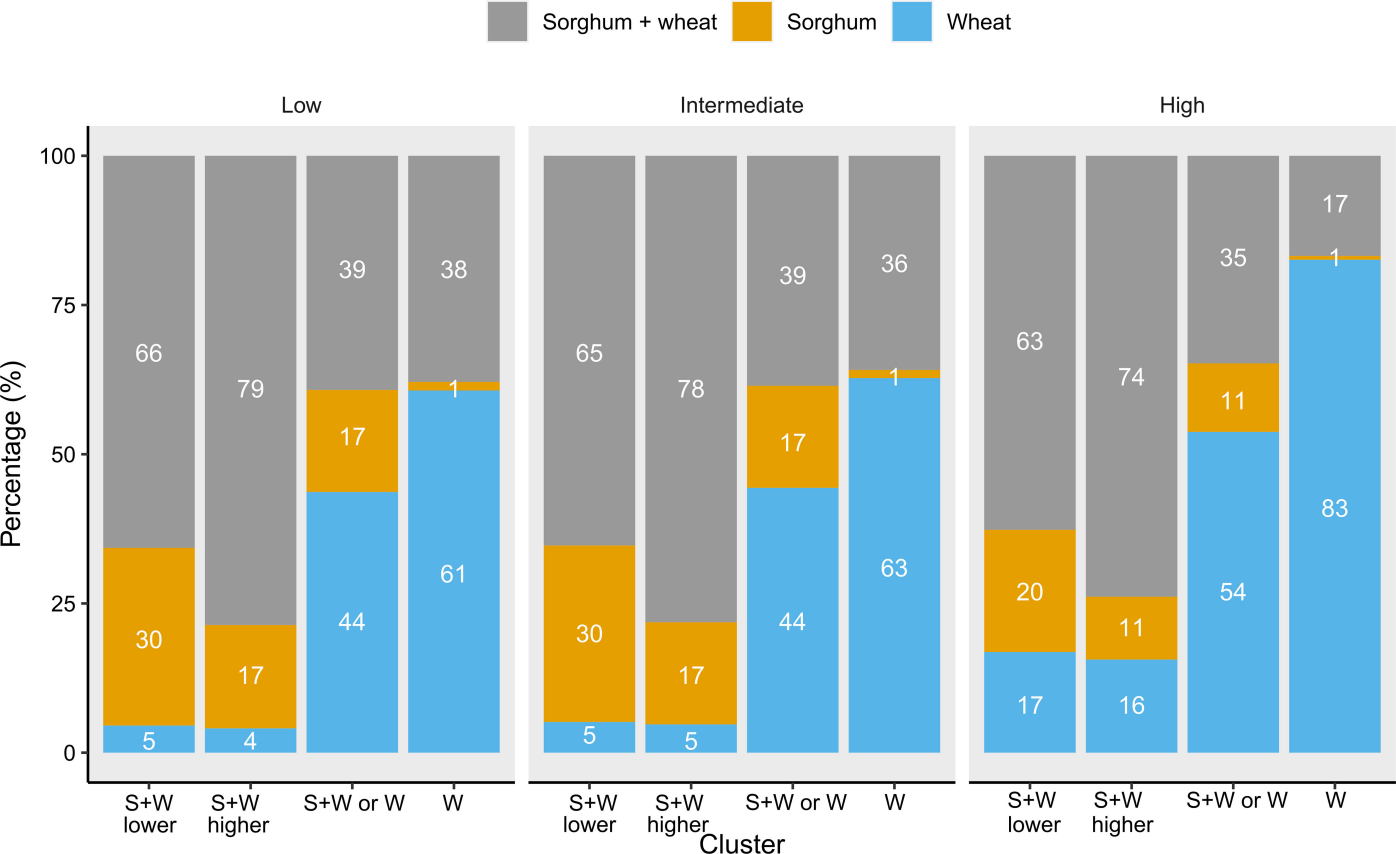
Frequency in which each rotation presented higher profits for each cluster and cost scenario. Low, intermediate, and high are the different cost scenarios. The colors represent each rotation. The bars represent each cluster for each cost scenario. S+W lower means the sorghum–wheat crop sequence presented higher profits and the income was lower, S+W higher means the sorghum–wheat crop sequence presented higher profits and the income was higher, S+W or W means that there is no clear difference between the sorghum–wheat crop sequence and wheat monocrop, and W means wheat monocrop presented higher profits.

In agreement with **Table 2**, the percentages of each crop sequence were similar when comparing the same clusters and cropping sequences in low- and intermediate-cost scenarios (**Figure 3**). In all the cost scenarios, for clusters S+W lower and S+W higher, the sorghum–wheat crop sequence presented frequencies higher than 60% and 70%, respectively. The difference between those clusters is in the percentage of sorghum monocrop (between 30% and 11%). In the low- and intermediate-cost scenarios, sorghum decreased from 30% to 17% from S+W lower to S+W higher, and in the high-cost scenario, sorghum decreased from 20% to 11%, respectively. In those clusters, wheat increased from 5% to 16% when the scenarios were low or intermediate and high, respectively. In cluster S+W or W, wheat was the cropping sequence with higher frequency across all cost scenarios, but the percentage was higher in high than in low and intermediate (54% vs. 44%). In the W cluster under all the cost scenarios, wheat had a higher profit in more than 61% of the total county × year combinations followed by the sorghum–wheat crop sequence (17%–38%). In this cluster, sorghum had the lower percentage (1%).

### 3.4 Environmental covariate analysis

**Figure 4:**
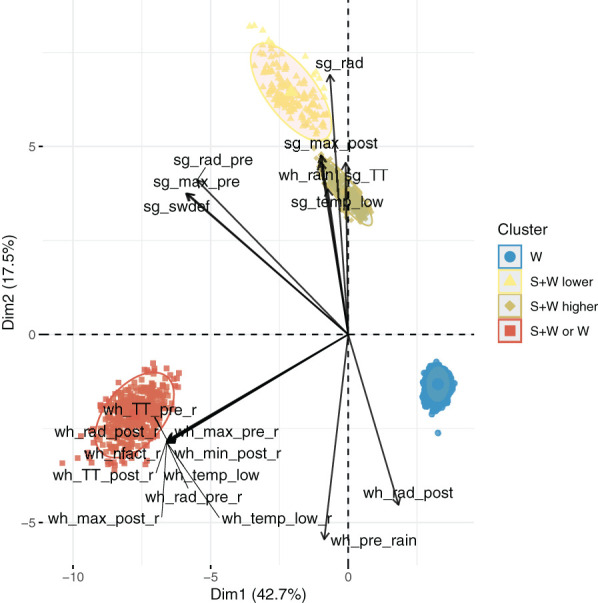
Principal component analysis (PCA) biplot of the four clusters with the vectors representing the environmental factors, and the colors represent each cluster. wh_max_post_r = average maximum temperature after wheat flowering in the sorghum–wheat crop sequence; wh_max_pre_r = average maximum temperature before wheat flowering in the sorghum–wheat crop sequence; wh_rad_pre_r = wheat seasonal cumulative solar radiation before wheat flowering in the sorghum–wheat crop sequence; wh_rad_post_r = wheat seasonal cumulative solar radiation after flowering in the sorghum–wheat crop sequence; wh_min_post_r = average minimum temperature after wheat flowering in the sorghum–wheat crop sequence; wh_TT_post_r = cumulative thermal time after wheat flowering in the sorghum–wheat crop sequence; wh_TT_pre_r = cumulative thermal time before wheat flowering in the sorghum–wheat crop sequence; wh_temp_low_r = number of days during the wheat crop with temperatures lower than 0°C in the sorghum–wheat crop sequence; wh_nfact_r = nitrogen stress during the whole wheat crop cycle in the sorghum–wheat crop sequence; sg_max_post = average maximum temperature after sorghum flowering in the sorghum–wheat crop sequence; sg_max_pre = average maximum temperature before sorghum flowering in the sorghum–wheat crop sequence; sg_rad = cumulative solar radiation during the whole sorghum crop cycle in the sorghum–wheat crop sequence; sg_TT = cumulative thermal time during the whole sorghum crop cycle in the sorghum–wheat crop sequence; sg_rad_pre = cumulative solar radiation before sorghum flowering in the sorghum–wheat crop sequence; sg_temp_low = number of days with temperatures lower than 10°C for sorghum in the sorghum–wheat crop sequence; sg_swdef = water deficit of sorghum crop in the sorghum–wheat crop sequence; wh_rad_post = cumulative solar radiation after wheat flowering in wheat monocrop; wh_temp_low = number of days during the wheat crop with temperatures lower than 0°C in wheat monocrop; wh_pre_rain = cumulative rain before wheat flowering in wheat monocrop; wh_rain = cumulative rain during the whole wheat crop cycle in wheat monocrop. The points stand for each simulation × year × cost scenario. S+W = cluster where sorghum–wheat crop sequence has better profits with lower income; S+W higher = sorghum–wheat crop sequence has better profits with higher income; S+W or W = there is no clear difference between the sorghum–wheat crop sequence and wheat monocrop; W = wheat monocrop with better profits.

The biplot captured 60% of the variability (**Figure 4**). Clusters S+W lower and S+W higher were closer to each other in the biplot and mainly related to sorghum variables. The variable seasonal solar radiation for sorghum crop (sg_rad) had the highest effect (longest vector), while maximum temperature after flowering (sg_max_post), seasonal thermal time (sg_TT), the number of days with temperature lower than 10°C for sorghum (sg_temp_low), and cumulative rain for wheat (wh_rain) were also closely related to both clusters. Radiation and maximum temperature before flowering and water deficit for sorghum crop (sg_rad_pre, sg_max_pre, and sg_swdef, respectively) were correlated with both clusters, but the angles between the vectors and the clusters were larger (**Figure 4**). The W cluster was affected by radiation after flowering (wh_rad_post) and cumulative rain before flowering for wheat monocrop (wh_pre_rain), but the angles were larger than those for the other clusters. Finally, cluster S+W or W was only affected by variables impacting wheat, which are wheat’s cumulative thermal time, radiation, and maximum temperature before flowering on the crop sequence (wh_TT_pre_r, wh_rad_pre_r, and wh_max_pre_r, respectively); radiation after flowering, cumulative thermal time, minimum, and maximum temperature after flowering (wh_rad_post_r, wh_TT_post_r, wh_min_post_r, and wh_max_post_r, respectively); nitrogen deficit and days with temperatures lower than 0°C and nitrogen deficit during wheat crop in the sorghum–wheat crop sequence (wh_nfact_r and wh_temp_low_r, respectively); and days with temperatures lower than 0°C (wh_temp_low) in wheat monocrop.

## 4 Discussion

This study provides new insights into the inclusion of sorghum as an alternative option for diversifying and intensifying the current wheat monocrops in the US Central Great Plains. New alternatives in this region are mainly limited due to limited rainfall (Hansen et al., 2012; Rosenzweig and Schipanski, 2019). However, our study highlights potential regions within Kansas with the opportunity to expand double cropping with the inclusion of early-maturing sorghum hybrids followed by a winter wheat crop, increasing farming profits. Previous studies have shown the benefits of a winter wheat–summer crop–fallow rotation versus a wheat–fallow rotation (Hansen et al., 2012; Nielsen et al., 2002), but to the extent of our knowledge, the sorghum–wheat crop sequence has not been explored yet until now.

Farmers decide their crop options based on profits, in addition to input costs and production problems linked to weeds, insects, and disease control (Socolar et al., 2021). More intensive and diverse cropping sequences are known to reduce weeds and break the cycle of pests and diseases (Liebman and Dyck, 1993; Rosenzweig et al., 2018; Wicks, 1984). A sorghum–wheat sequence is expected to increase water use efficiency and reduce farmers’ reliance on subsidies and off-farm income (Dhuyvetter et al., 1996; Hansen et al., 2012; Nielsen et al., 2005). In Kansas, areas with greater precipitation and higher temperatures have higher profits when wheat monocropping is replaced by a sorghum–wheat sequence (Kansas Office of the State Climatologist Kansas Climate, 2024; Miller et al., 2021; Staggenborg et al., 2008; Tack et al., 2015). Conversely, in regions with lower temperatures and similar precipitation, there was no clear difference between having sorghum–wheat or wheat monocrop as the main options. The regions with wheat monocrop clusters tended to have lower rainfall and lower wheat yields compared to the rest of the state, which may be due to lower rainfall (lftt and Gaku, 2024). These results are consistent with other studies comparing different wheat–fallow crop sequences (Anderson et al., 1999; Bushong et al., 2012; Massigoge et al., 2024).

Higher cropping intensities can positively impact crop water availability by improving ground cover and soil infiltration, e.g., improved rainfall harvest efficiency, as well as reducing evaporative losses during fallows (Holman et al., 2020; Massigoge et al., 2024; Simão et al., 2023). In Kansas, low rainfall, especially in the west, poses challenges for farmers (Deines et al., 2019; “Kansas Office of the State Climatologist · Kansas Climate,” 2024; Stone et al., 2006). In addition, over time, the western part of the state became dryer relative to the eastern region, with the latter presenting more precipitation (Lin et al., 2017). Water deficit during the sorghum crop affected both clusters where the sorghum–wheat crop sequence had the higher profits. For the sorghum crop, the timing of water deficit (with similar intensity and duration) produces a differential impact on yield, with a reduction in grain number when the stress occurs around flowering and an impact on grain weight when a similar stress takes place during the grain filling period (Prasad et al., 2008). Consistent with Johnson and Kanemasu (1982), rainfall during wheat crop also impacted these clusters. Water deficit has a large impact on wheat yield, even more so when combined with high-temperature stress during the reproductive period (Nicolas et al., 1984). Radiation during sorghum crop highly affected the S+W lower and S+W higher clusters, aligning with previous findings on the positive effect of this factor on crop productivity (Pepper and Prine, 1972). The S+W or W cluster was only influenced by environmental variables of the wheat crop, mostly in the sorghum–wheat crop sequence. Minimum and maximum temperatures before and after flowering can affect wheat’s yield by affecting several factors including crop growth and vernalization. Another variable related to temperature is thermal time, which implies the duration of the crop stages (Xiao et al., 2017). Lastly, radiation can have a positive effect on yield by increasing photosynthesis (Demotes-Mainard and Jeuffroy, 2004; Thorne and Wood, 1987) for wheat. This indicated that the wheat crop plays a more significant role in the S+W or W cluster, where there is no clear advantage between sorghum–wheat and wheat monocrop. Lastly, for the W cluster, the factors affecting it were radiation during the grain filling period. According to Shimoda and Sugikawa (2020), lower radiation during this period, especially at the beginning, decreases grain weight. Future gains in wheat yield will need to not only support increases in the intercepted radiation to improve biomass production but also accompany further enhancements in radiation use efficiency (Reynolds et al., 2012). Rainfall during the vegetative period also affected the cluster, being a higher rainfall positive for crop production (Johnson and Kanemasu, 1982). Lastly, for both wheat and sorghum grown under rainfed conditions, water supply defines the attainable upper limit for yield, even when radiation and temperature are critical factors for determining the potential productivity (van Ittersum and Rabbinge, 1997). The limitations of this study include i) the lack of extensive datasets to validate crops in a sorghum–wheat crop sequence rather than the independent monocrops; ii) wheat was considered the main crop, and the duration of the sorghum crop was shortened to match the recommended planting date of wheat, yet sorghum was not tested as the main crop in the sequence; and iii) broader genetic variability was not tested. In addition, including other alternative winter crops such as canola (*Brassica napus* L.) could be more attractive due to the current demand for oil and biofuels. Future steps could be focused on integrating new field datasets on this rotation, quantifying regional impacts for expanding this more intensified crop sequence, and testing other rotations such as wheat–sorghum or canola–sorghum to evaluate changes in both productivity (including seed quality parameters, protein, and oil) and profit over time.

## 5 Conclusion

We showed that in the wetter regions, a sorghum–wheat crop sequence outperformed monocrops, while in the drier environments, wheat–fallow monocrops remained the most profitable option for farmers. That is, across Kansas, the sorghum–wheat crop sequence was a superior choice (with varying levels of profitability) in the southeast and south-central regions (30% of the counties), the wheat monocrop in the western regions occupied 40% of the counties, and in the rest of the state, we detected no significant differences between the sorghum–wheat crop sequence and the wheat monocrop. Therefore, a possibility for expanding more intensified and diversified cropping systems (reducing the risk) is available for farmers across this region. For the main environmental drivers, water deficits, radiation, and extreme temperatures are the major weather factors limiting yields for wheat and sorghum under the current rainfed agricultural systems. A map delineating these geo-clusters provides a practical tool for farmers, suggesting optimal rotations designed for specific regions within Kansas, with the potential to transfer a similar approach to a regional scale. Specifically, in certain areas, intensifying rotations promises higher profits and improved water use efficiency. Conversely, other areas may see greater financial gains from maintaining a wheat monocrop system.

## Data Availability

The original contributions presented in the study are included in the article/**Supplementary Material**. Further inquiries can be directed to the corresponding authors.
