# Supplementary material for: Intensifying cropping sequences in the US Central Great Plains: an in silico analysis of a sorghum–wheat sequence
Source: Front Plant Sci. 2025 May 30;16:1525128. doi: 10.3389/fpls.2025.1525128 (PMC12165406; doi:10.3389/fpls.2025.1525128)
Supplement: Supplementary file 1 [file DataSheet1.docx]

**Supplementary Data**

**Supplementary Figures**


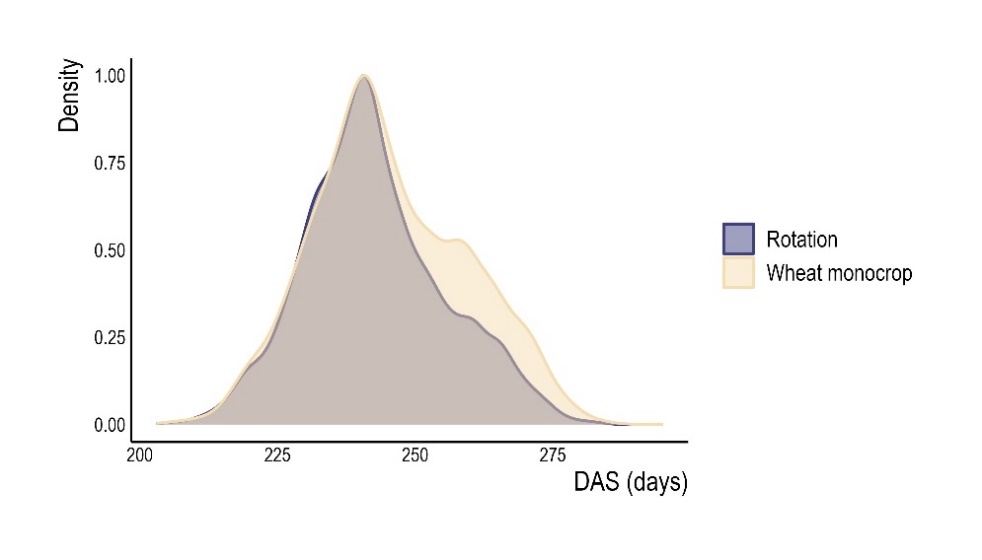


A


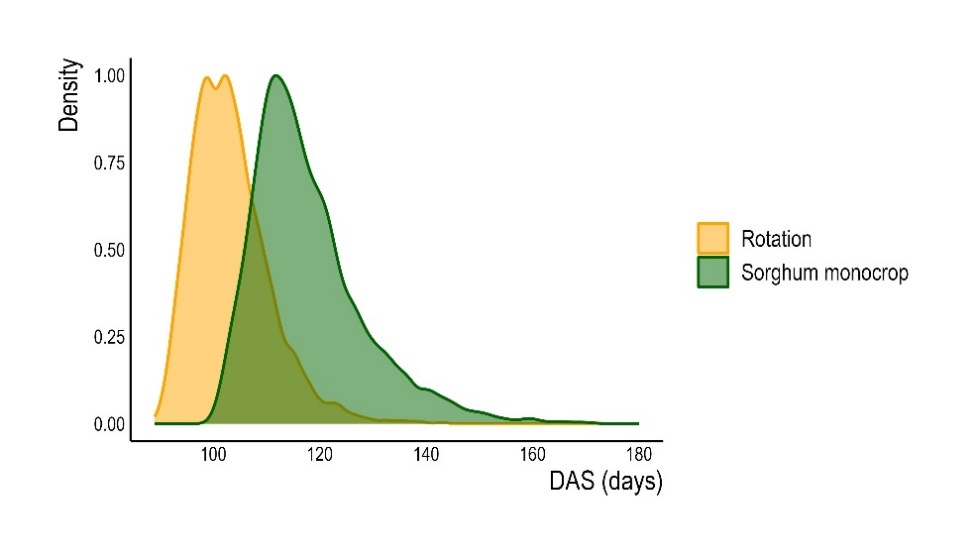


B

Supplementary Figure 1. Days to maturity in days after sowing for sorghum and wheat in the sorghum-wheat crop sequence and in monoculture. A- Wheat, monocrop in barley white and in sorghum-wheat crop sequence in dark blue. B- Sorghum, monocrop in green, and in sorghum-wheat crop sequence in orange.


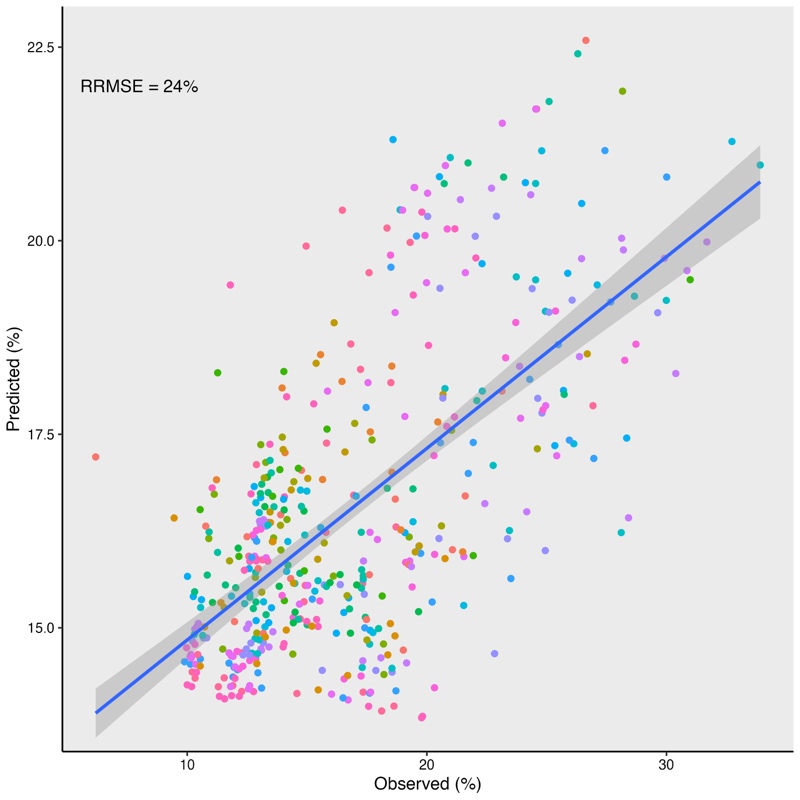


Supplementary Figure 2. Dry-down model validation. The different colors represent the different hybrids tested.


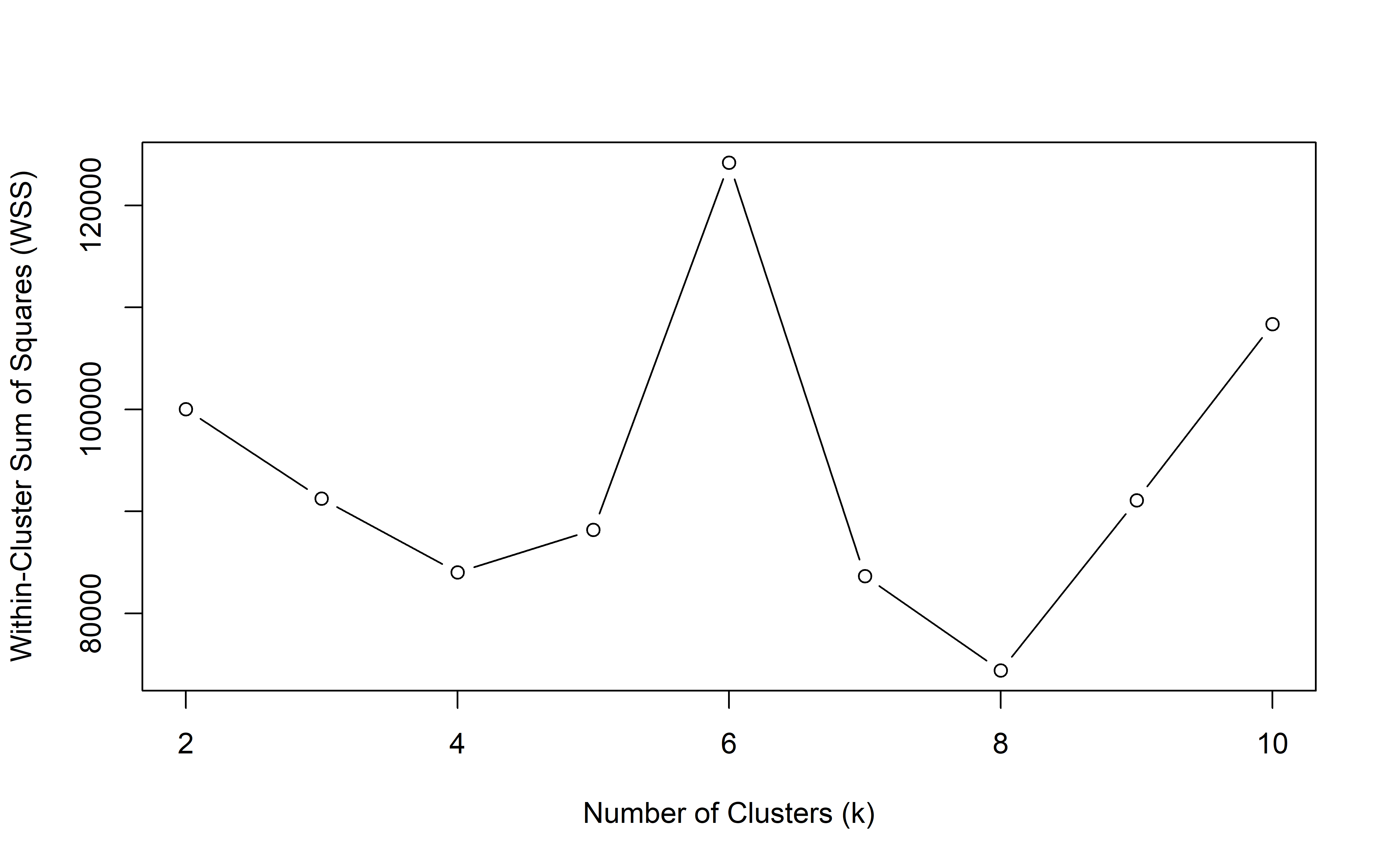


Supplementary Figure 3. The optimal number of clusters.


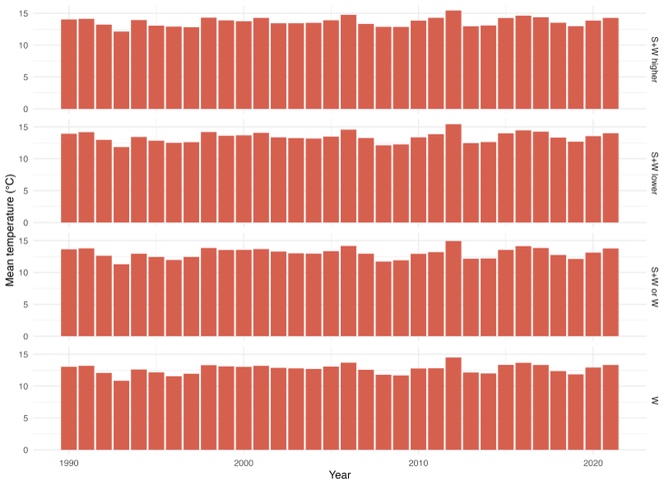

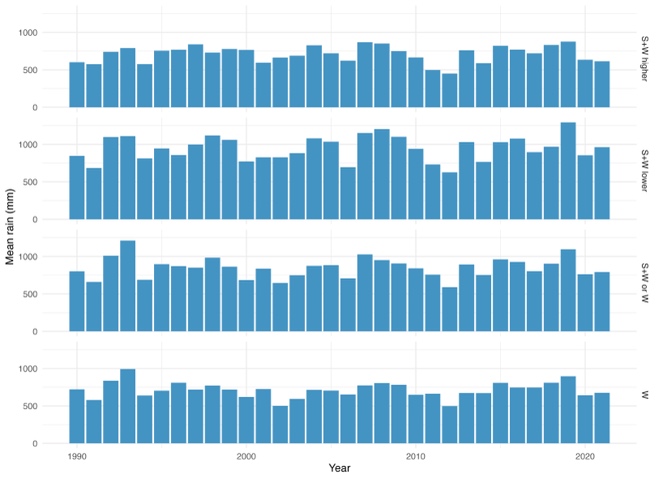


Supplementary Figure 4. Interannual temperature (left) and rain (right) for each cluster from 1990 to 2020.

**Supplementary Tables**

Supplementary Table 1. Planting date and rate for wheat and sorghum crop across Kansas state.

|  | Zone | Wheat | Sorghum |
| --- | --- | --- | --- |
|  |  |  |  |
| Planting date | 1 | Sept 10–30 | May 15–June 10 |
|  | 2 | Sept 15–Oct 20 | May 15–June 20 |
|  | 3 | Sept 25–Oct 20 | May 15–June 20 |
|  | 4 | Oct 5–25 | May 1–15-June 5–25 |
| planting rate  (pl m ^-2^) | western | 186 |  |
|  | central | 205 |  |
|  | eastern | 247 |  |
|  | Less than 20 |  | 5.9 |
|  | 20-26 |  | 8.8 |
|  | 26-32 |  | 11 |
|  | more than 32 |  | 17.3 |
|  |  |  |  |

Supplementary Table 2. Sorghum Buster hybrid coefficients

| Description | Coefficient | Value |
| --- | --- | --- |
|  |  |  |
| tt_emerg_to_endjuv | tt_emerg_to_endjuv | 100 |
| photoperiod_crit1 | photoperiod_crit1 | 11.5 |
| photoperiod_crit2 | photoperiod_crit2 | 13.5 |
| photoperiod_slope | photoperiod_slope | 11.5 |
| tt_endjuv_to_init | tt_endjuv_to_init | 160 |
| tt_flag_to_flower | tt_flag_to_flower | 170 |
| tt_flower_to_start_grain | tt_flower_to_start_grain | 80 |
| tt_flower_to_maturity | tt_flower_to_maturity | 761 |
| tt_maturity_to_ripe | tt_maturity_to_ripe | 1 |
| dm_per_seed | dm_per_seed | 0.00083 |
| maxGFRate | maxGFRate | 0.09 |
| look up table for canopy height | x_stem_wt | 0 80 |
| plant canopy height"> | y_height | 0 2000 |
| Largest leaf multiplier | aX0 | 0.786 |
| Largest Leaf Area Slope | aMaxS | 46.312 |
| Intercept for Largest leaf calculation | aMaxI | 321.13 |
|  |  |  |

Supplementary Table 3. Sorghum pioneer_s34 hybrid coefficients

| Description | Coefficient | Value |
| --- | --- | --- |
|  |  |  |
| tt_emerg_to_endjuv | tt_emerg_to_endjuv | 100 |
| photoperiod_crit1 | photoperiod_crit1 | 12.3 |
| photoperiod_crit2 | photoperiod_crit2 | 14.6 |
| photoperiod_slope | photoperiod_slope | 25 |
| tt_endjuv_to_init | tt_endjuv_to_init | 115 |
| tt_flag_to_flower | tt_flag_to_flower | 100 |
| tt_flower_to_start_grain | tt_flower_to_start_grain | 30 |
| tt_flower_to_maturity | tt_flower_to_maturity | 695 |
| tt_maturity_to_ripe | tt_maturity_to_ripe | 1 |
| dm_per_seed | dm_per_seed | 0.00083 |
| maxGFRate | maxGFRate | 0.09 |
| look up table for canopy height | x_stem_wt | 0 80 |
| plant canopy height"> | y_height | 0 2000 |
|  |  |  |

Supplementary Table 4. Wheat Larry hybrid coefficients.

| Description | Coefficient | Unit | Value |
| --- | --- | --- | --- |
|  |  |  |  |
| Sensitivity to vernalization | vern_sens | 1(lowest)-5(highest) | 2.4 |
| Sensitivity to photoperiod | photop_sens | 1(lowest)-5(highest) | 2.03 |
| Thermal time to end of juvenile | tt_end_of_juvenile | °Cd | 454 |
| Thermal time to floral initiation | tt_floral_initiation | °Cd | 570 |
| Thermal time to grain filling | tt_start_grain_fill | °Cd | 596 |
|  |  |  |  |

Supplementary Table 5. Seed and fertilizer costs, and grain prices for sorghum and wheat from 2014 to 2022. The yearly grain prices, seed and fertilizer costs were used to classify the years into cost scenarios. Classification was done in base of the sum of seed and ertilizer for both crops. Inter stands for Intermediate. Fert stands for fertilizer.

|  |  | 2022 | 2021 | 2020 | 2019 | 2018 | 2017 | 2016 | 2015 | 2014 |
| --- | --- | --- | --- | --- | --- | --- | --- | --- | --- | --- |
|  | Classification | High | Inter | Low | Inter | Low | Low | Inter | High | High |
| Sorghum | Seed (USD ha^-1^) | 32.4 | 29.9 | 28. | 28.6 | 32.7 | 33.1 | 33.5 | 31.5 | 31.3 |
|  | Fert (USD ha^-1^) | 199.1 | 106.4 | 89.7 | 99.7 | 73.8 | 77.0 | 85.6 | 101.2 | 102.2 |
|  | Price (USD ton^-1^) | 275.4 | 215.3 | 174.0 | 129.2 | 123.7 | 121.8 | 104.9 | 136.3 | 136.7 |
| Wheat | Seed (USD ha^-1^) | 26.8 | 24.8 | 24.3 | 24.3 | 24.7 | 24.2 | 29.3 | 31.2 | 30.4 |
|  | Fert (USD ha^-1^) | 163. | 86.1 | 73.2 | 82.1 | 76.1 | 73.9 | 70.8 | 83.7 | 90.1 |
|  | Price (USD ton^-1^) | 359.9 | 234.6 | 168.9 | 179.9 | 197.2 | 155.6 | 145.0 | 207.1 | 265.2 |
|  | Sum | 170.8 | 100.1 | 87.5 | 95.0 | 83.9 | 84.3 | 88.8 | 100.3 | 102.9 |
|  |  |  |  |  |  |  |  |  |  |  |

Supplementary Table 6. Average seed and fertilizer costs and grain prices for sorghum and wheat under the three cost scenarios. The prices and costs on the table were used in the profit calculations.

|  | Classification | High | Intermediate | Low |
| --- | --- | --- | --- | --- |
| Sorghum | Seed (USD ha^-1^) | 31.8 | 30.7 | 31.6 |
|  | Fertilizer (USD ha^-1^) | 134.2 | 97.3 | 80.2 |
|  | Price (USD ton^-1^) | 182.9 | 149.9 | 139.9 |
| Wheat | Seed (USD ha^-1^) | 29.5 | 26.2 | 24.4 |
|  | Fertilizer (USD ha^-1^) | 112.5 | 79.7 | 74.5 |
|  | Price (USD ton^-1^) | 277.5 | 186.5 | 174.0 |
|  |  |  |  |  |

Supplementary Table 7. Average yield values for each crop within each rotation and cluster.

| Cluster | Wheat yield monocrop | Sorghum yield monocrop | Wheat yield rotation | Sorghum yield rotation |
| --- | --- | --- | --- | --- |
|  |  |  |  |  |
| S+W or W | 2553 | 2501 | 962 | 2193 |
| S+W lower | 3200 | 4581 | 1185 | 4246 |
| S+W higher | 3689 | 4938 | 1539 | 4550 |
| W | 3930 | 3140 | 1703 | 2995 |
|  |  |  |  |  |
