# Supplementary figures and images for: Intensifying cropping sequences in the US Central Great Plains: an in silico analysis of a sorghum–wheat sequence
Source: Front Plant Sci. 2025 May 30;16:1525128. doi: 10.3389/fpls.2025.1525128 (PMC12165406; doi:10.3389/fpls.2025.1525128)

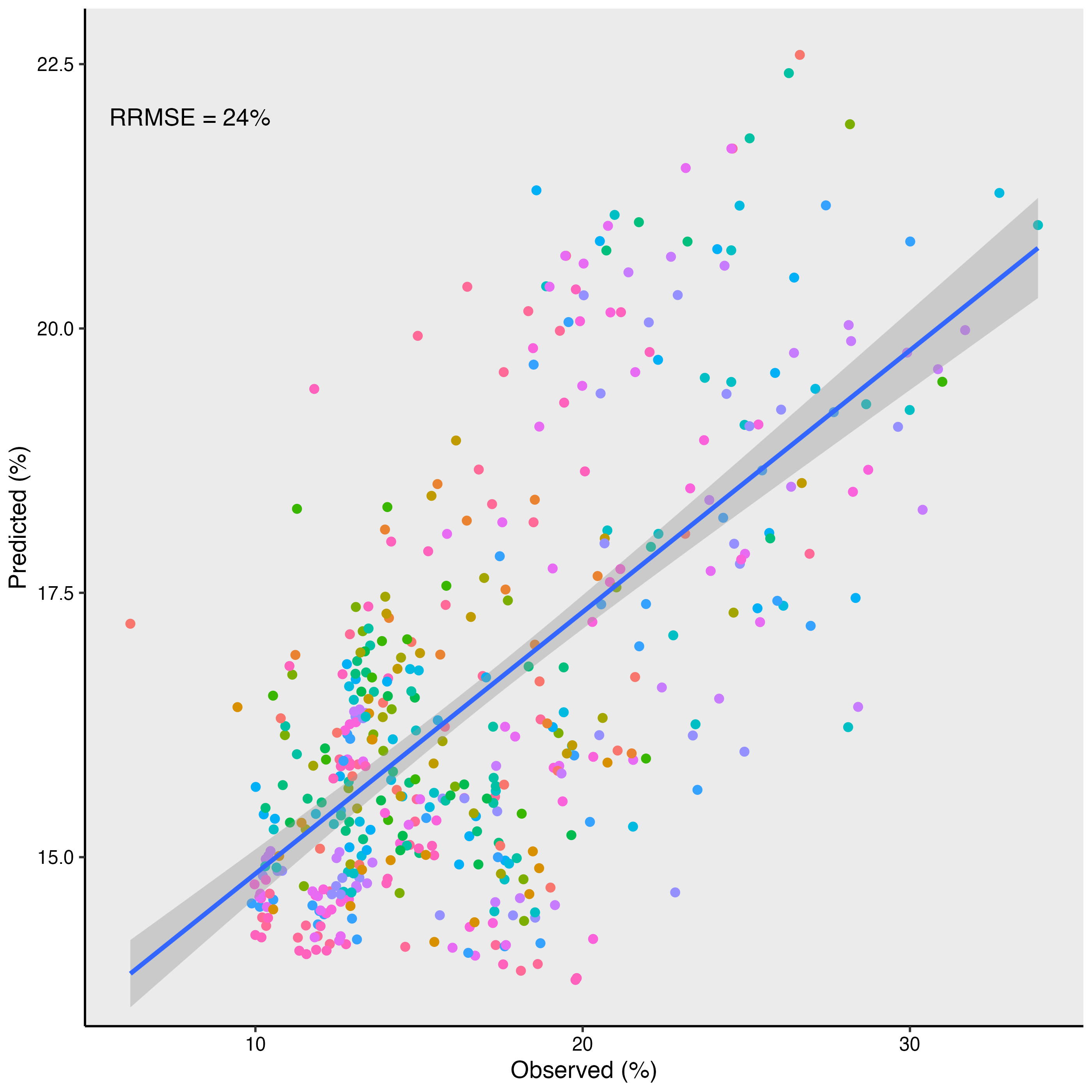

Supplement: Supplementary file 2 [file Image1.jpeg]

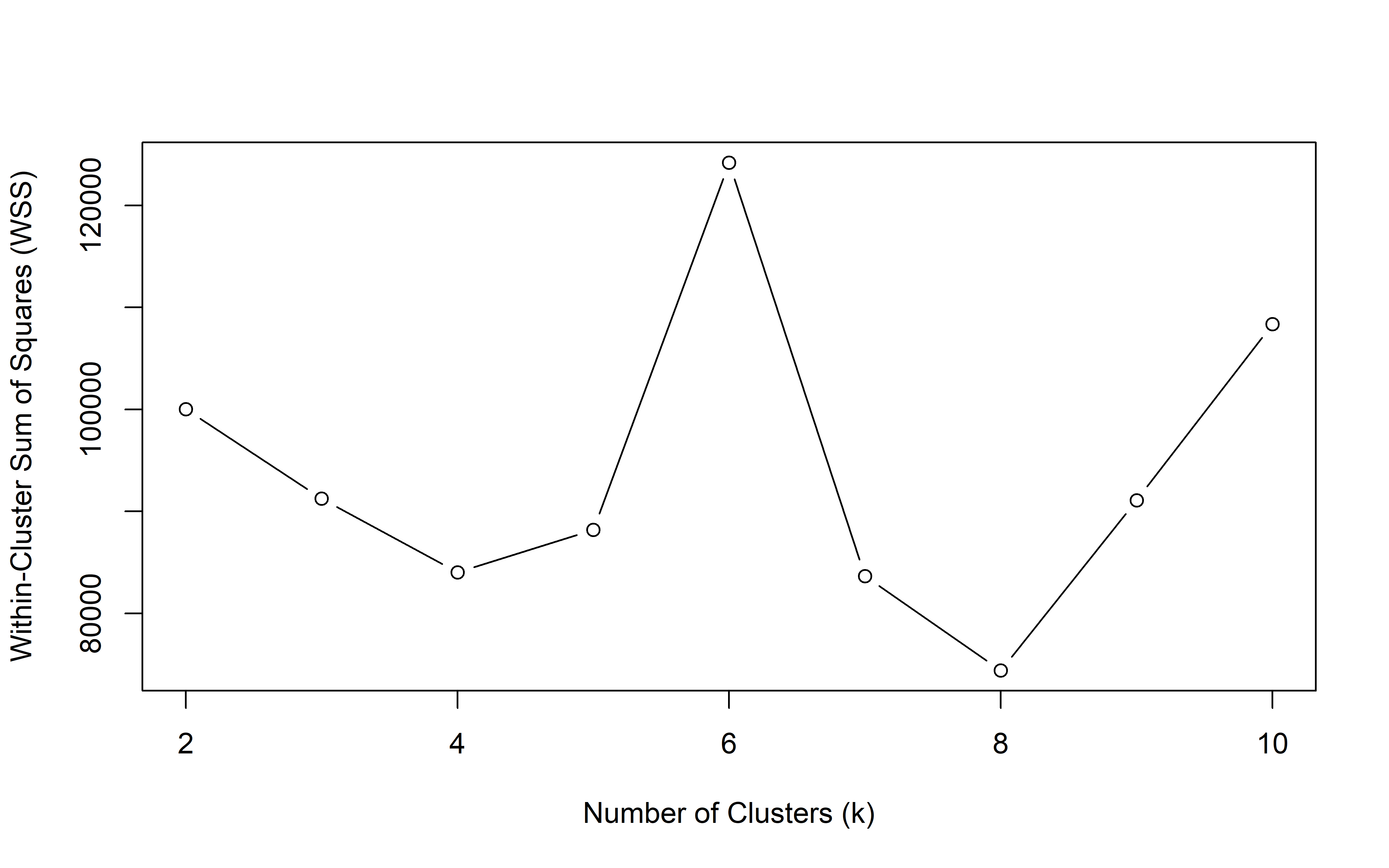

Supplement: Supplementary file 3 [file Image2.jpeg]
